# ONECUT2 is a druggable driver of luminal to basal breast cancer plasticity

**DOI:** 10.1007/s13402-024-00957-3

**Published:** 2024-05-31

**Authors:** Irene Zamora, Mirian Gutiérrez, Alex Pascual, María J. Pajares, Miguel Barajas, Lillian M. Perez, Sungyong You, Beatrice S. Knudsen, Michael R. Freeman, Ignacio J. Encío, Mirja Rotinen

**Affiliations:** 1 Department of Health Sciences, Public University of Navarre, Pamplona, Navarre Spain; 2 IdiSNA, Navarre Institute for Health Research, Pamplona, Navarre Spain; 3 Department of Urology, Cedars-Sinai Medical Center, Los Angeles, CA USA; 4 Department of Medicine, University of California Los Angeles, Los Angeles, CA USA; 5 Department of Pathology, University of Utah, Salt Lake City, UT USA

**Keywords:** ONECUT2, Transcription factor, Estrogen receptor, Cell plasticity, Heterogeneity, Breast cancer

## Abstract

**Purpose:**

Tumor heterogeneity complicates patient treatment and can be due to transitioning of cancer cells across phenotypic cell states. This process is associated with the acquisition of independence from an oncogenic driver, such as the estrogen receptor (ER) in breast cancer (BC), resulting in tumor progression, therapeutic failure and metastatic spread. The transcription factor ONECUT2 (OC2) has been shown to be a master regulator protein of metastatic castration-resistant prostate cancer (mCRPC) tumors that promotes lineage plasticity to a drug-resistant neuroendocrine (NEPC) phenotype. Here, we investigate the role of OC2 in the dynamic conversion between different molecular subtypes in BC.

**Methods:**

We analyze OC2 expression and clinical significance in BC using public databases and immunohistochemical staining. *In vitro*, we perform RNA-Seq, RT-qPCR and western-blot after OC2 enforced expression. We also assess cellular effects of OC2 silencing and inhibition with a drug-like small molecule *in vitro* and *in vivo*.

**Results:**

OC2 is highly expressed in a substantial subset of hormone receptor negative human BC tumors and tamoxifen-resistant models, and is associated with poor clinical outcome, lymph node metastasis and heightened clinical stage. OC2 inhibits ER expression and activity, suppresses a gene expression program associated with luminal differentiation and activates a basal-like state at the gene expression level. We also show that OC2 is required for cell growth and survival in metastatic BC models and that it can be targeted with a small molecule inhibitor providing a novel therapeutic strategy for patients with OC2 active tumors.

**Conclusions:**

The transcription factor OC2 is a driver of BC heterogeneity and a potential drug target in distinct cell states within the breast tumors.

**Graphical Abstract:**

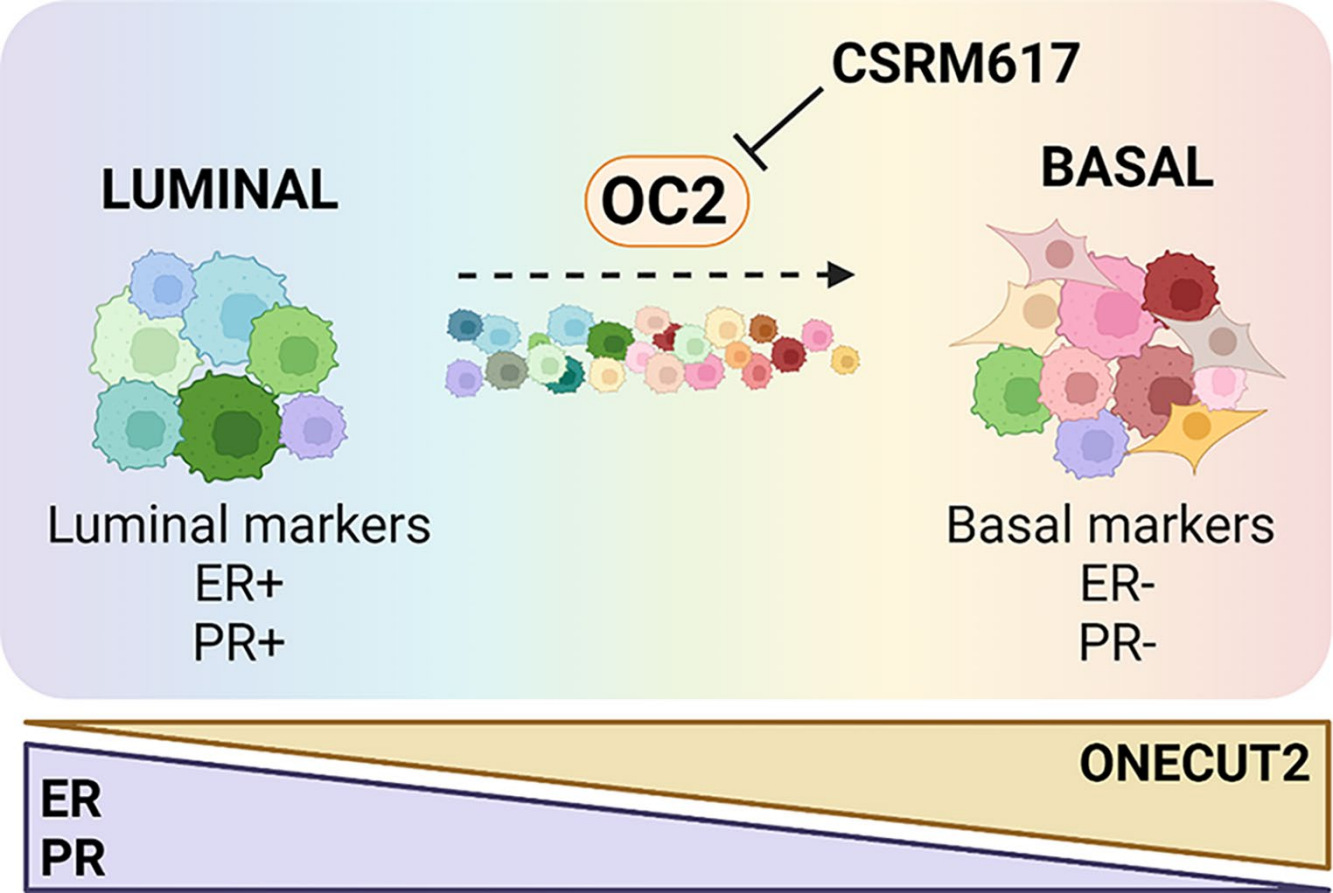

**Supplementary Information:**

The online version contains supplementary material available at 10.1007/s13402-024-00957-3.

## Introduction

Breast cancer (BC) is a heterogeneous disease. The presence of specific markers in BC (estrogen receptor (ER), progesterone receptor (PR) and human epidermal receptor 2 (HER2)) has long been used to define subtypes for the allocation of individual cases to specific categories [1]. With the onset of microarray gene expression profiling of large sets of tumors in the last decades, BC is classified into at least four major molecular subtypes: luminal A and luminal B (both ER and PR positive), human epidermal growth factor receptor 2 positive (HER2+) and basal-like (also called triple-negative breast cancer (TNBC)) [2, 3]. Each of these subtypes has different risk factors for incidence, risk of disease progression, response to treatment and preferential organ sites of metastasis [4, 5]. However, it is important to consider that heterogeneity within individual tumors is significantly more frequent than the assignments by ER, PR, and HER2 status or any molecular subtype. For instance, it is recommended that ER and PR assays be considered positive if there are at least 1% positive tumor nuclei in the sample [6], implying that, in many cases, the majority of tumor cells display cancer markers inconsistent with the assigned subcategory of the tumor [1]. At the molecular level, the best studied clinical subtype of BC is TNBC where at least six molecular subtypes have been described [7] and where substantial spatial subclonal heterogeneity is found compared to other BC subtypes [8, 9]. Added to the complexity of intratumoral diversity is the dynamic interconversion between different molecular subtypes. “Receptor conversion” in the course of disease progression in BC has been frequently reported, especially between primary tumors and paired metastasis within a patient [10]. Inter- and intratumoral ER and PR loss may thus be an important additional hallmark of endocrine therapy response failure. Identifying the molecular drivers underlying the evolving tumor heterogeneity in BC is crucial to develop new drugs that can counteract the formation of new phenotypes in an individual patient’s tumor and thereby delay the development of drug resistance, especially in the metastatic disease.

Here, we investigate the role of the transcription factor ONECUT2 (OC-2/OC2/HNF6β; hereafter, OC2) in the dynamic conversion between different molecular subtypes in BC. OC2 is a member of the ONECUT class of transcription factors with important developmental functions [11, 12] that has been associated with tumor cell proliferation, angiogenesis, and metastasis [13, 14]. We previously showed that OC2 is a master regulator protein of metastatic castration-resistant prostate cancer (mCRPC) that promotes lineage plasticity to a drug-resistant neuroendocrine (NEPC) phenotype [15]. In this paper we demonstrate that OC2 is involved in the “receptor conversion” from luminal/hormone-receptor positive tumors to basal/hormone-resistant/TNBC, thus contributing to tumor heterogeneity. We further show that in BC OC2 acts as a survival factor and can be inhibited with a drug-like small molecule that can be used to target multiple cell states within the breast tumors.

## Materials and methods

### Clinical outcome analysis

**Fig. 1:**
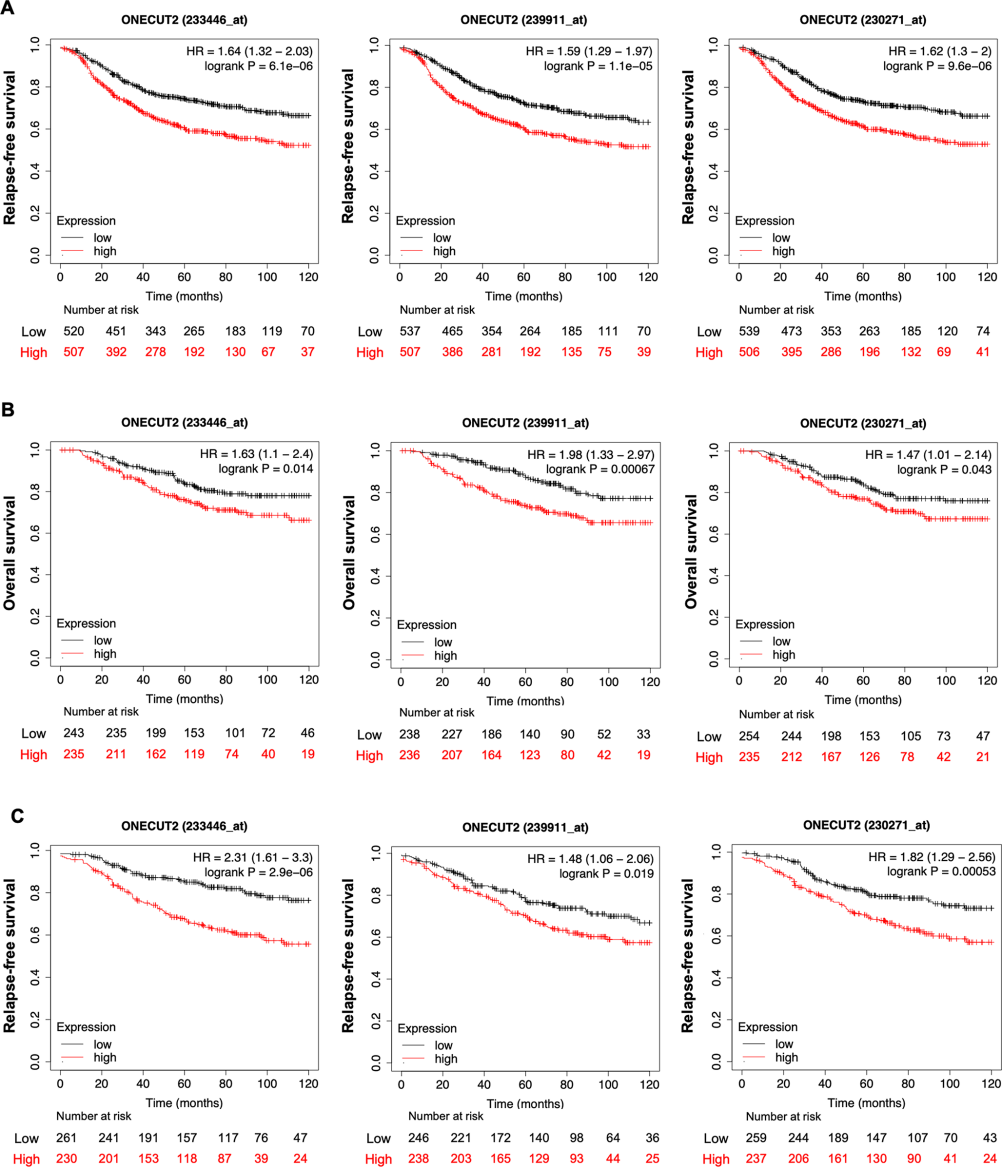
OC2 expression is associated with poor prognosis in BC patients. **A**, **B** Association of relapse-free survival (**A**) and overall survival (**B**) with OC2 expression using three different probes (239911_at, 233446_at and 230271_at). **C** Association of relapse-free survival with OC2 expression in luminal A BC patients (probes: 239911_at, 233446_at and 230271_at) according to the St. Gallen classification. For (**A**–**C**) patients were divided according to OC2 mRNA expression levels using Q1 vs. Q4 quartiles as cut-off. Log-rank Test. For every comparison, *P* < 0.05 was considered significant

We studied the relationships between the OC2 expression and relapse-free survival (RFS) and overall survival (OS) in BC shown in Fig. 1 and Supplementary Fig. 1. The Kaplan-Meier Plotter (http://kmplot.com) tool was used to perform Kaplan-Meier analysis and log-rank test. This database includes gene expression data and survival information from 7830 patients with BC, downloaded from the Gene Expression Omnibus (GEO), European Genome-phenome Archive (EGA) and The Cancer Genome Atlas (TCGA) [16]. Patients were divided according to OC2 mRNA expression levels (probes: 239911_at, 233446_at and 230271_at) using the median (Supplementary Fig. 1) and Q1 vs. Q4 quartiles (Fig. 1 and Supplementary Fig. 1E) as cut-off. For every comparison, *P* < 0.05 was considered significant.

### Breast cell lines culture

BC cell lines were obtained from the American Type Culture Collection. MCF-7, BT-474, T-47D, 4T1 and MDA-MB-231 were grown in RPMI-1640 media (Gibco) supplemented with 10% fetal bovine serum (FBS) and 1% penicillin/streptomycin. 184B5 cells were grown in DMEM media (Gibco) supplemented with 10% FBS and 1% penicillin/streptomycin. All cell lines tested negative for mycoplasma contamination.

### Antibodies and reagents

Anti-OC2, rabbit, polyclonal (Sigma, HPA057058, lot no. A104348) (1:100) was used for the immunohistochemistry (IHC) studies; anti-OC2, rabbit, polyclonal (Sigma, HPA057058, lot no. A104348) (1:1000) and anti-OC2, rabbit, polyclonal (Proteintech, 21916-1-AP, lot no. 00114937) (1:1000) were used for western blot. The following antibodies were used for western blot: anti-β-actin, mouse, monoclonal (Sigma #A5441, lot no. 0000126950) (1:2000); anti-Estrogen Receptor alpha (D8H8), rabbit, monoclonal (Cell Signaling, #8644, lot no. 8) (1:1000); anti-Progesterone Receptor A/B (D8Q2J) XP, rabbit, monoclonal (Cell Signaling, #8758, lot no. 7) (1:1000); anti-CD44 (156-3 C11), mouse, monoclonal (Invitrogen #MA5-13890, lot no. ZA393694) (1:1000); anti-TGFB1, rabbit, monoclonal (Invitrogen #MA5-15065, lot no. ZA4181581) (1:1000); anti-rabbit IgG, HRP-linked (Cell Signaling #7074, lot no. 28) (1:5000); anti-mouse IgG, HRP-linked (GE-Healthcare NA931, lot no. 17,271,474) (1:5000). The reagents used were: doxycycline hyclate (Sigma #D9891), β-Estradiol (Sigma #E4389), tamoxifen (Sigma #T2859) and CSRM617 (Sigma #SML2608).

### Lentiviral constructs

Lentiviral particles were generated by cotransfection of the pCMV-Delta-8.2 (Addgene #12263) packaging plasmid and the pCMV-VSVG plasmid (Addgene #8454) into HEK-293 cells using Lipofectamine 2000 (Invitrogen). Medium was changed every 24 h and the 48 h and 72 h supernatants were filtered through a 0.45 μm PVDF filter syringe for transduction of the recipient cells. The constitutive OC2 overexpression plasmid was purchased from Origene (Lenti-ORF clone of ONECUT2 (mGFP-tagged)-Human one cut homeobox 2 # RC211951L4). As a control for the experiments the empty vector pLenti-C-mGFP-P2A-Puro (Origene # PS100093) was used. The inducible OC2 overexpression plasmid was obtained by cloning the full length OC2 cDNA (NM_004852) and the enhanced GFP (E-GFP) into the pCW57-MCS1-P2A-MCS2 (Neo) (Addgene # 89180). Cells were subsequently transduced in the presence of 10 µg mL^− 1^ of polybrene (Millipore) and selected on 5 µg mL^− 1^ puromycin (Gibco) or 1 mg mL^− 1^ geneticin G418 (Gibco) to create the stable lines. The experiments to knockdown OC2 with shRNAs were performed as previously described [15].

### siRNA transfection

OC2 (SR306292) siRNA pools and Universal scrambled negative control siRNA duplexes (Origene) were used according to manufacturers’ instructions. Briefly, cells were transfected with siRNA (100 nM) for 48 h with Lipofectamine RNAiMAX Reagent (Invitrogen).

### Apoptosis quantification

Cells were collected and stained with APC-Annexin V and 7-Aminoactinomycin D (7-AAD) (BioLegend) according to the manufacturers’ instructions. Apoptotic cells were detected by flow cytometry using a Beckman Coulter CytoFLEX Flow Cytometer (excitations at 488, 638, 405, 355, 561, 808 nm) and data were analyzed with CytoFLEX CytExpert Software v. 2.4.9.28. Cells were gated by FSC-A x SSC-A to exclude debris and then by FSC-H x FSC-W following SSC-H x SSC-W to exclude cell doublets.

### RNA-sequencing and gene set enrichment analysis

Total RNA was extracted using the RNeasy Kit (Qiagen). QC analysis, library preparations and sequencing were performed at the CIMA Genomics Core (Center for Applied Medical Research, University of Navarre) and data normalization and processing at the CIMA Bioinformatics Platform. RNA-Sequencing (RNA-Seq) data analysis was performed using the following workflow: (1) the quality of the samples was verified using FastQC software (https://www.bioinformatics.babraham.ac.uk/projects/fastqc/) and the trimming of the reads with Trimmomatic [17]; (2) the alignment of reads against the human reference genome (GRCh38) was performed using STAR [18]; (3) gene expression quantification using read counts of exonic gene regions was carried out with featureCounts [19]; (4) the gene annotation reference was GENCODE V42 [20]; and (5) differential expression statistical analysis was performed with R/Bioconductor (https://www.R-project.org/). Gene expression data was normalized with edgeR [21] and Voom [22]. After quality assessment and outlier detection using R/Bioconductor, a filtering process was performed. Genes with read counts lower than 4 in more than the 50% of the samples of all the studied conditions were considered as not expressed in the experiment under study. LIMMA (Linear Models for Microarray Data) [22] was used to identify the genes with significant differential expression between experimental conditions. Further functional and clustering analyses and graphical representations were performed using R/Bioconductor, MSigDB [23] and clusterProfiler [24]. Data are available in GEO database with the accession number GSE242541.

### OC2 expression profile

Differential OC2 gene/protein expression patterns in normal and corresponding cancerous tissues were analyzed using cBioportal [25, 26], UALCAN [27, 28], OncoDB [29] and CANCERTOOL [30] servers. For single-cell RNA-Seq (scRNA-Seq) analysis, epithelial cells with cell type information were selected from the metadata in the Single-Cell Portal [31] and OC2 expression was normalized using log normalization. For the OC2 dependency analysis in different BC cell lines, ONECUT2 RNAi Screening Data from the DepMap portal [32] was selected and grouped by molecular subtypes.

### Quantitative PCR

Total RNA was extracted (RNeasy Kit, Qiagen) and subjected to One-Step Real Time RT-PCR in a CFX-Connect Real Time PCR Detection System (Bio-Rad) with AgPath-ID One-Step RT-PCR Reagents (Applied Biosystems) and the TaqMan probes for ONECUT2 (Hs00191477_m1), ESR1 (Estrogen receptor alpha) (Hs01046816_m1), PGR (Progesterone receptor) (Hs01556702_m1), PRLR (Hs01061477_m1), MYC (Hs00153408_m1), GREB1 (Hs00536409_m1), TFF1 (Hs00907239_m1), PDZK1 (Hs00275727_m1), GATA3 (Hs00231122_m1), MET (Hs01565584_m1), TGFB1 (Hs00998133_m1) and CD44 (Hs01075864_m1). Data were analyzed using the 2^−ΔΔCT^ method. ACTB, TUBB and GAPDH TaqMan probes (Hs01060665_g1, Hs00742828_s1 and Hs02758991_g1, respectively) were used for normalization.

### Western blot analysis

Cell lysates were separated on 4–20% SDS-PAGE (Bio-Rad Laboratories) and transferred to PVDF membranes. The membranes were blocked with 5% BSA and subsequently incubated with the pertinent primary antibody overnight. Membranes were washed with PBST (0.1% Tween-20) and incubated with an HRP-conjugated secondary antibody. After washing with PBST, the protein bands were detected by chemiluminescence (SuperSignal West Pico PLUS Chemiluminescent Substrate, ThermoFisher Scientific).

### Proliferation assays

Cell viability was assayed by crystal violet staining. Briefly, cells (250,000 cells per well in six-well plates) were seeded and transfected as indicated above. Cells were then fixed in 4% formaldehyde, washed, and subsequently stained with 0.1% crystal violet. Quantitation was performed by extracting the crystal violet dye with 10% acetic acid and measuring the absorbance at 595 nm on a Sunrise microplate reader coupled to the Magellan software (Tecan).

### Dose-response curves and IC50

Cells were plated at a density of 1,000 cells per well, in triplicate, in a 96-well plate. The next day, T0 readings were collected and cells were exposed to compound CSRM617, tamoxifen, or vehicle (1% DMSO) at 2-fold concentration ranging from 100 µM to 0.2 µM. Viability was measured 48 h post-treatment using CellTiter-Glo Luminescent Cell Viability Assay (Promega). Dose-response curves and IC50 values were generated using GraphPad Prism version 9 Software. The synergistic effect of CSRM617 and tamoxifen or cisplatin was predicted using SynergyFinder 3.0, a software for analyzing dose-response matrix data of drug combinations [33]. Cells were treated with CSRM617 at a final concentration of 100, 50, 25, 12.5 or 6.25 µM and with tamoxifen or cisplatin at 100, 50, 25, 12.5 or 6.25 µM for 48 h.

### IHC

The expression of OC2 was assessed using immunohistochemical staining. Briefly, a BC tissue microarray (BR1503f, US Biomax Inc.) containing 50 breast invasive ductal carcinomas (IDC) was deparaffinized and rehydrated. Endogenous peroxidase activity was quenched using 3% H_2_O_2_ for 10 min and microwave antigen retrieval was carried out with EDTA buffer (EnVision™ FLEX Target Retrieval Solution, High pH, Dako Omnis) twice for 10 min. Non-specific binding sites were blocked with 5% normal goat serum in TBS-Tween (Wash buffer, Dako) for 30 min. Sections were incubated with anti-OC2 antibody (1:100; HPA057058 Sigma) overnight at 4 °C. Detection was performed with ENVISION HRP system (Dako). The peroxidase activity was visualised with diaminobenzidine. Finally, sections were washed, lightly counterstained with haematoxylin, dehydrated and mounted. The specificity of the OC2 antibody was demonstrated by western blot analysis and IHC of cell lines expressing different levels of the protein. Negative controls were performed by omission of the primary antibody. Staining scores were established by semiquantitative analysis as previously described [34]. The extension of the staining was scored as the percentage of positive cells (0–100%) and the intensity of the staining was assessed using a 4-value scoring system (0 = below the level of detection, 1 = weak, 2 = moderate and 3 = strong). A final H score was calculated by adding the product of the percentage cells stained at a given staining intensity (0–100) and the staining intensity (0–3). The median value of all H scores was chosen as the cut-off point to separate low from high OC2 expressing tumours. Representative images of low, intermediate, and high OC2 expression in breast carcinomas are shown in Supplementary Fig. 7.

### Animal studies

All experimental protocols and procedures were approved by the Institutional Animal Care and Use Committee (IACUC) of the University of Navarre (CEEA) under the protocol CEEA/055 − 22. All relevant ethical regulations, standards, and norms were rigorously adhered to. Female BALB/c mice (6–9 weeks old) were purchased from Charles River Laboratories and maintained under specific pathogen-free conditions. To assess the effect of compound CSRM617 in vivo, 100,000 4T1 cells were resuspended in a total volume of 100 µL PBS and injected subcutaneously into the flanks of BALB/c mice. Mice bearing tumors with a mean volume of 50 mm^3^ were randomly divided into vehicle control (2.5% DMSO in PBS) or compound CSRM617 (50 mg·kg^− 1^) groups and subjected to intraperitoneal injection daily. Tumor volume was measured and calculated using the formula V = 1/2 · length · width^2^. Serum biochemistry analysis in mice plasma was performed at the CIMA Biochemical and Metabolic Analysis Core using a Cobas c-311 (Roche Diagnostics).

### Statistics and reproducibility

Statistically significant data for *in vitro* and *in vivo* assays were assessed by two-tailed Student’s t-test. The normality of the data was assessed using a Shapiro-Wilk test. Groups with normal distribution followed a t-test. Non-normal samples were analyzed using a Wilcoxon two-tailed rank-sum test. For comparison of more than two groups, a residual test was performed to study normality and a Levene test to assess homoscedasticity. When both requirements were met, one-way analysis of variance (ANOVA) corrected for multiple comparisons using Tukey’s posthoc test was performed. Kruskal-Wallis and Dunn’s tests were applied otherwise. Survival analyses were done using the log-rank test. For every comparison, *P* < 0.05 was considered statistically significant and *P* < 0.01, highly significant. We used Excel and the R package (R version 4.1.2) for all statistical tests. Data visualization was implemented using GraphPad (Version 9) and BioRender (www.biorender.com).

## Results

### OC2 expression is associated with poor clinical outcome in BC

The developmental transcription factor OC2 has been identified as a master regulator of aggressive prostate cancer variants that can be inhibited with a small molecule to suppress established metastasis in preclinical models [15]. OC2 expression has been shown to be significantly associated with poor clinical outcome in cancer types other than prostate, including breast, lung, gastric, clear cell renal, colon and brain [15]. Consistent with the possibility of a role for OC2 in human BC, an extended Kaplan-Meier analysis using three different probes shows that high expression of OC2 mRNA is associated with worse relapse-free survival (RFS) and overall survival (OS) with all probes tested (Fig. 1A, B and Supplementary Fig. 1A–D). This association is also found in luminal A tumors suggesting a prognostic biomarker role of OC2 in this BC subtype (Fig. 1C and Supplementary Fig. 1E–H).

### OC2 expression is associated with lymph node metastasis and clinical stage

**Fig. 2:**
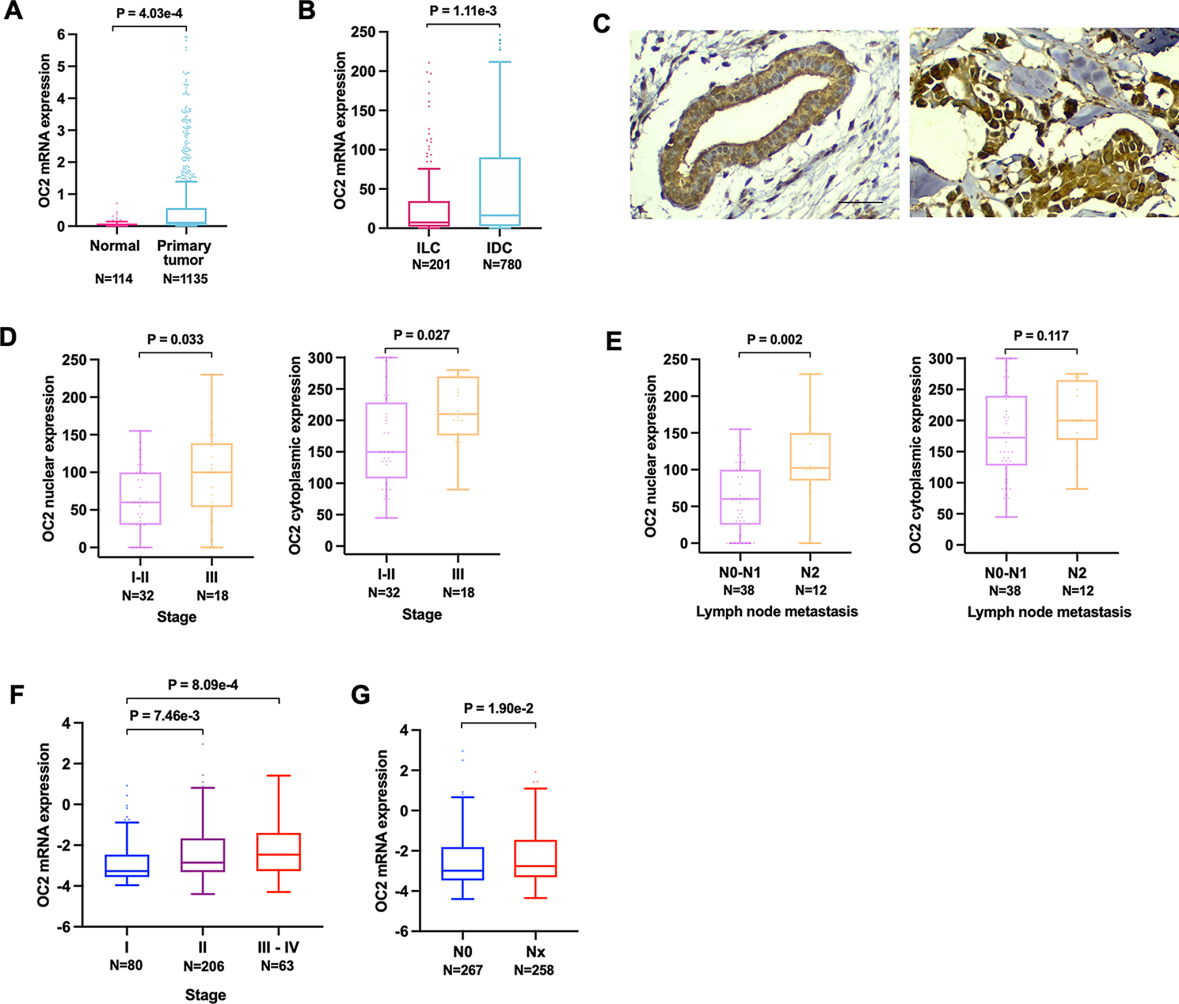
Association between OC2 expression and clinical pathological parameters in patients with BC. **A** OC2 mRNA expression in normal breast tissue compared to BC primary tumors from the TCGA cohort. **B** OC2 mRNA expression in invasive ductal carcinoma (IDC) and invasive lobular carcinoma (ILC) from the TCGA cohort. For (**A**, **B**) Wilcoxon two-tailed rank-sum test *P*-values are shown. **C** Immunohistochemical staining of OC2 in benign (left) and breast cancer tissue (right). OC2, brown. Scale bar, 50 μm. **D**, **E** Association of stage (**D**) and lymph node metastasis (**E**) with OC2. Boxplots of intensity levels of nuclear (left) and cytoplasmic (right) OC2 expression assessed by IHC using a BC TMA are shown. The boxes show the 25–75th percentile range and the center line is the median. Whiskers extend from the minimum and maximum values. *P*-values were obtained from Wilcoxon two-tailed rank-sum test. **F**, **G** Association of stage (**F**) and lymph node metastasis (**G**) with OC2 mRNA expression from BC tumors from the TCGA cohort. The boxes show the 25–75th percentile range and the center line is the median. Whiskers show 1.5 times the interquartile range (IQR) from the 25th or 75th percentile values. For (**F**) Kruskal-Wallis and Dunn’s tests *P*-values are shown. For (**G**) Wilcoxon two-tailed rank-sum test *P*-values are shown. N0 = no spread to regional lymph nodes, Nx = N1, N2 and N3 grouped. For every comparison, *P* < 0.05 was considered significant

The World Health Organization classifies breast epithelial tumors into multiple histologic types. Among these, invasive ductal carcinoma (IDC) is the most common type, accounting for 70–80% [35] and followed by invasive lobular carcinoma (ILC). In the TCGA, OC2 mRNA is elevated in primary tumors compared with benign breast tissue (Fig. 2A) and in IDC compared with ILC (Fig. 2B). In the METABRIC breast cancer cohort [36–38] OC2 mRNA is also significantly higher in IDC compared to ILC and other histologic types of breast tumors (Supplementary Fig. 2A). Quantitative analysis of OC2 immunostaining intensity, using a BC tissue microarray (TMA) containing benign breast tissue, intraductal carcinoma and IDC, showed nuclear and cytoplasmic OC2 protein staining in epithelial cells of the ducts in normal tissue and in tumor cells (Fig. 2C). We further analyzed the relationship between changes in the levels of OC2 expression in tumors and clinicopathological features including age, cancer stage and TNM stage. OC2 nuclear and cytoplasmic staining were positively correlated with tumor stage and lymph node metastasis (Fig. 2D, E). Consistent with this, in the TCGA cohort, OC2 mRNA increased gradually with tumor stage and nodal metastasis status (Fig. 2F, G). In the METABRIC cohort OC2 expression is also significantly associated with histologic grade (Supplementary Fig. 2B). Of note, the promoter methylation of OC2 is downregulated with grade (Supplementary Fig. 2C) suggesting that decreased methylation levels of OC2 might be an underlying indicator which reflects clinical characteristics of BC patients.

### OC2 negatively regulates the ER and its transcriptional program

**Fig. 3:**
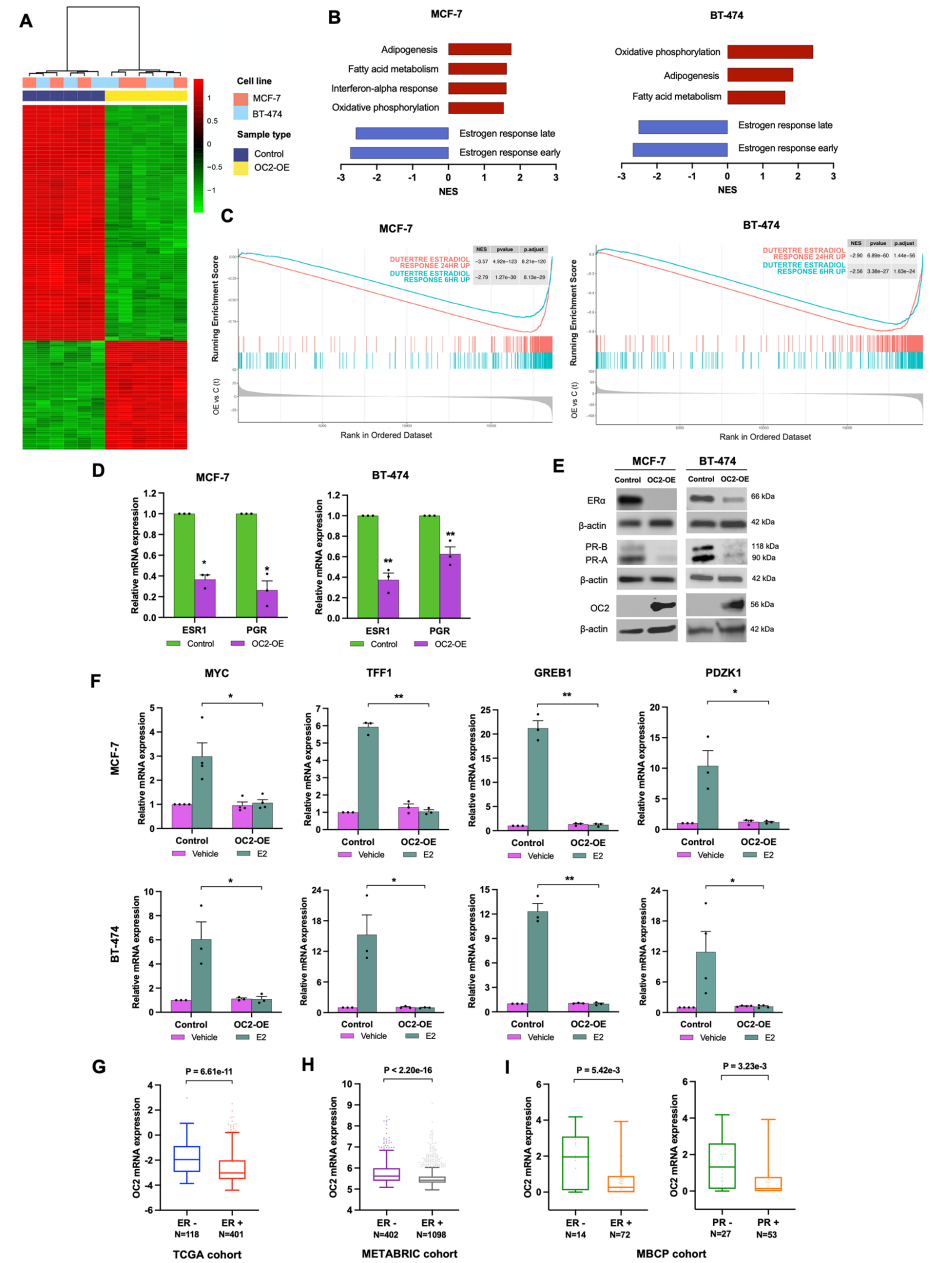
OC2 suppresses ER expression and its transcriptional program. **A** Heatmap depicts the overlap between differentially expressed genes significantly perturbed (|log FC| ≥ 1.5 and adjusted *P*-value < 1e-10) by ectopic OC2 expression in MCF-7 and BT-474 cells. Three independent RNA-Seq experiments were performed per condition. **B** Enriched cellular processes by OC2 perturbed genes. MSigDB Hallmark Gene Sets enriched in OC2 upregulated (red bars) and downregulated (blue bars) genes in MCF-7 (left) and BT-474 (right) cells that overexpress OC2. NES = normalized enrichment score. **C** GSEA plots of two estrogen activity signatures obtained after 6 and 24 h treatment with E2 [39] showing enrichment of ER target genes in MCF-7 (left) and BT-474 (right) cells that overexpress OC2. Three independent RNA-Seq experiments were performed. **D**, **E** ER and PR mRNA (**D**) and protein (**E**) expression in MCF-7 and BT-474 cells after enforced expression of OC2. Representative blots from three independent experiments. PR-A and PR-B = progesterone receptor isoforms. qRT-PCR results were normalized using β-actin. The mean + S.E.M. from three independent experiments is shown. Unpaired two-tailed Student’s t-test, *P < 0.05, **P < 0.01. **F** MYC, GREB1, PDZK1 and TFF1 mRNA levels in MCF-7 (upper panel) and BT-474 (lower panel) cells with enforced OC2 expression, treated for 24 h with 10 nM E2 or vehicle. The mean + S.E.M. from three to four independent experiments is shown. Unpaired two-tailed Student’s t-test was used for statistical analysis, *P < 0.05, **P < 0.01. **G**, **H** Expression of OC2 mRNA in ER-negative vs. ER-positive patients in the (**G**) TCGA and (**H**) METABRIC cohorts. **I** Expression of OC2 mRNA in ER-negative vs. ER-positive (left) and in PR-negative vs. PR-positive (right) patients in the MBCP cohort. For (**G**, **H**) the boxes show the 25–75th percentile range and the center line is the median. Whiskers show 1.5 times the IQR from the 25th or 75th percentile values. For (**I**) the boxes show the 25–75th percentile range and the center line is the median. Whiskers extend from the minimum and maximum values. Wilcoxon two-tailed rank-sum test *P*-values are shown. For every comparison, *P* < 0.05 was considered significant

OC2 protein is expressed in the luminal B BT-474 cell line, in the metastatic triple-negative MDA-MB-231 BC cells, in weakly metastatic luminal A MCF-7 BC cells and at detectable but substantially lower levels in luminal A T-47D and nontumorigenic 184B5 cells (Supplementary Fig. 3A, B). To study the role of OC2 in aggressive BC we performed RNA-Seq in MCF-7 and BT-474 after enforced constitutive expression of OC2 (Fig. 3A). OC2-induced genes were enriched in pathways related to metabolism (e.g. adipogenesis, fatty acid metabolism, oxidative phosphorylation) and immune response (interferon alpha response) (Fig. 3B), whereas OC2-repressed genes were enriched in pathways corresponding to ER activity (both, estrogen response early and late hallmark genes) (Fig. 3B). These data suggest that OC2 interacts with the ER transcriptional program. To explore this further, we next performed gene set enrichment analysis (GSEA) with a 322-gene set of ER targets in an estrogen response signature [39] (genes up-regulated in MCF-7 cells after 24 h of estradiol (E2) treatment) using the transcriptome datasets obtained from OC2 overexpression. Enforced OC2 expression significantly repressed the majority of ER target genes (253/322, *P* = 8.21e-120 for MCF-7; 235/323, *P* = 1.44e-56 for BT-474) (Fig. 3C). A similar result was observed when analyzing another ER activity signature obtained after 6 h treatment with E2 [39] (120/226, *P* = 8.13e-29 for MCF-7; 117/227, *P* = 1.63e-24 for BT-474) (Fig. 3C) and temporal estrogen response metasignatures included in The EstroGene Database [40] (Supplementary Fig. 3C).

The above evidence suggests that OC2 suppresses ER activity. Among the top-100 most significantly repressed genes in the RNA-Seq dataset (|log FC| ≥ 1.5 and adjusted *P*-value < 1e-10), we found the estrogen receptor alpha gene (ESR1) (Supplementary Tables 1 and 2). Consistent with this, when OC2 expression was enforced in MCF-7 and BT-474 cells, ER mRNA and protein were downregulated (Fig. 3D, E). In accordance with the marked loss of ER, expression of the progesterone receptor (PGR) was significantly suppressed at the mRNA level when OC2 is overexpressed (Fig. 3D). We also observed downregulation of PRα and PRβ at the protein level (Fig. 3E). MYC, GREB1 (Growth Regulating Estrogen Receptor Binding 1), TFF1 (Trefoil Factor 1) and PDZK1 (PDZ Domain Containing 1) are classic regulatory targets of the ER [41, 42]. Ectopic expression of OC2 abolished the E2 induced transcription of these genes in MCF-7, BT-474 and T-47D cells (Fig. 3F and Supplementary Fig. 3D).

The above results imply that OC2 can operate under conditions of suppressed ER activity. Consistent with this, in the TCGA cohort, OC2 expression is significantly increased in ER negative BC tumors (Fig. 3G). Similarly, in the METABRIC cohort and in the Metastatic Breast Cancer project (MBCP) cohort OC2 mRNA levels are significantly higher in tumors where ER expression is suppressed (Fig. 3H, I). In the MBCP, OC2 expression is also significantly higher in PR negative tumors (Fig. 3I). In these cohorts, OC2 promoter methylation is increased in the ER positive tumors (Supplementary Fig. 3E, F) suggesting that loss of DNA methylation might be a prerequisite of increase in OC2 gene activity.

### OC2 promotes luminal to basal transition in BC cells

**Fig. 4:**
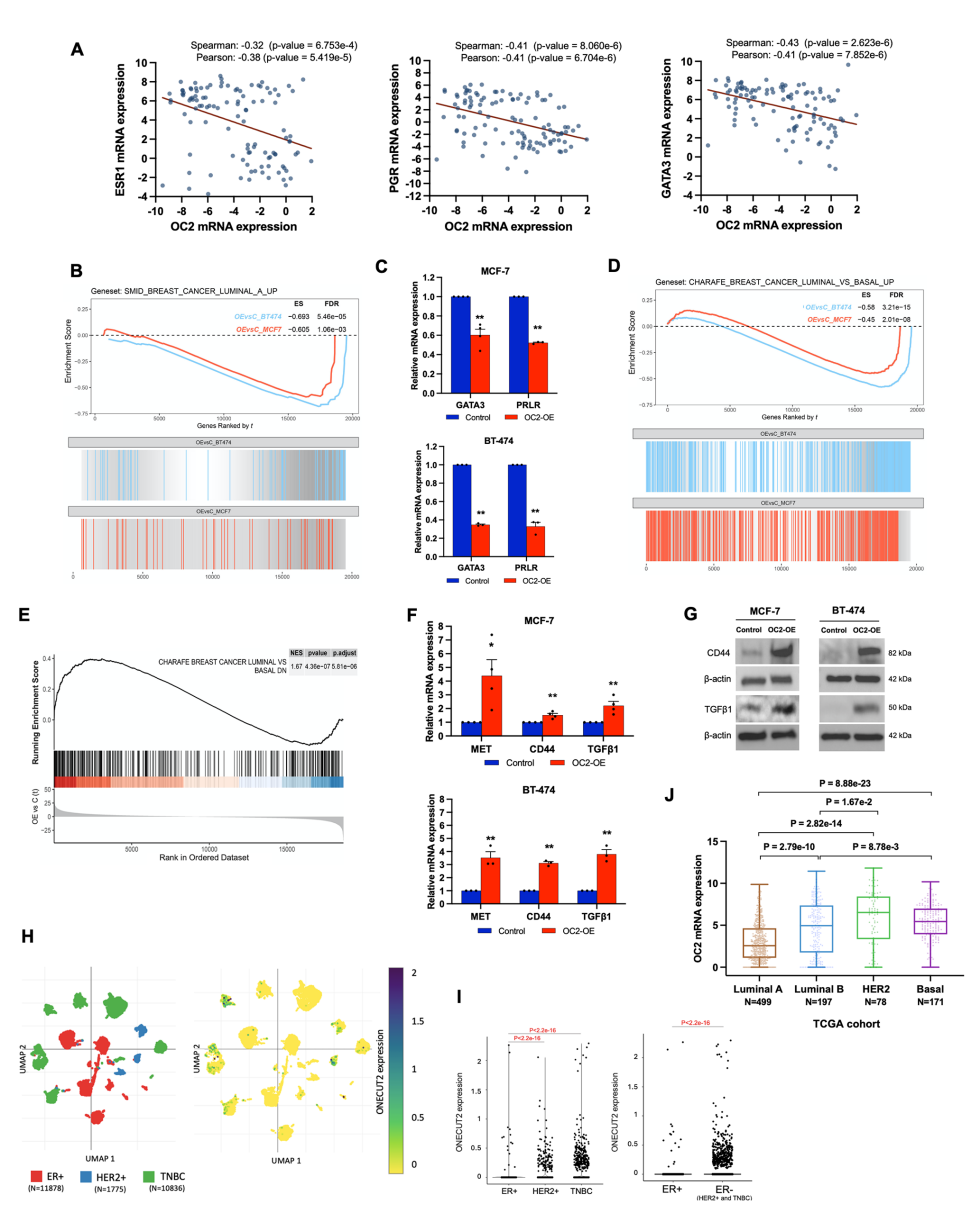
OC2 drives luminal to basal transition. **A** Inverse correlation between OC2 expression and ESR1 (left), PGR (middle) and GATA3 (right) expression in the CPTAC cohort. **B** GSEA plot showing negative enrichment of a luminal A signature [4] in MCF-7 and BT-474 cells with enforced OC2 expression. Three RNA-Seq experiments were performed per condition. **C** Expression of the luminal markers GATA3 and PRLR in MCF-7 (upper panel) and BT-474 (lower panel) cells after enforced OC2 expression. qRT-PCR results were normalized using β-actin. The mean + S.E.M. from three independent experiments is shown. Unpaired two-tailed Student’s t-test, *P < 0.05, **P < 0.01. **D** GSEA plot showing negative enrichment of genes up-regulated in luminal-like BC cell lines compared to the basal-like ones [44] in MCF-7 and BT-474 cells with enforced OC2 expression. **E** GSEA plot showing positive enrichment of genes down-regulated in luminal-like BC cell lines compared to the basal-like ones [44] in MCF-7 cells with enforced OC2 expression. For (**D**, **E**) three RNA-Seq experiments were performed per condition. **F**, **G** Expression of basal/mesenchymal markers (MET, CD44 and TFGB1) in MCF-7 and BT-474 cells after enforced OC2 expression. For (**F**) qRT-PCR results were normalized using β-actin. The mean + S.E.M. from three independent experiments is shown. Unpaired two-tailed Student’s t-test, *P < 0.05, **P < 0.01. For (**G**) representative blots from two independent experiments are shown. **H** Uniform Manifold Approximation and Projection (UMAP) plots illustrating BC subtypes (left) and OC2 expression (right) in BC epithelial cells. **I** The violin plots with data points depict OC2 expression levels in distinct BC subtypes. Wilcoxon two-tailed rank-sum test *P*-values are shown. For (**H**, **I**) data were obtained from the Single-Cell Portal [31]. **J** The boxplot shows OC2 mRNA expression in different BC subtypes. Data obtained from the TCGA cohort. The boxes show the 25–75th percentile range and the center line is the median. Whiskers extend from the minimum and maximum values. Kruskal-Wallis and Dunn’s tests *P*-values are shown. For every comparison, *P* < 0.05 was considered significant

The observation that OC2 represses ER and PR suggests that OC2 may play a role in the emergence of basal properties in luminal BC. Luminal BC cells are characterized by positive ER and/or PR expression. This type of cell also shows high expression of a panel of luminal-associated genes/proteins including luminal keratins (KRT8/18/19), transcription factors such as GATA3, FOXA1 or MYB and secretory proteins (e.g. TFF1, TFF3) [43]. In TCGA ER, PR and GATA3 are in the top-5 negatively correlated proteins with OC2 (Supplementary Table 3). ESR1, PGR and GATA3 mRNAs are also found consistently negatively correlated with OC2 expression across different cohorts (Fig. 4A and Supplementary Fig. 4A, B). Among the top-100 most significantly repressed genes in our RNA-Seq data (|log FC| ≥ 1.5 and adjusted *P*-value < 1e-10) we found at least 8 BC luminal markers (including ESR1, GATA3, TFF1, TFF3, MYB, XBP1, MUC1, CRABP2) [43]. These results are also consistent with a significant repression of up-regulated genes in luminal A and luminal B published signatures in the transcriptome dataset obtained from OC2 overexpression, as well as a significant de-repression of down-regulated genes in the luminal B signature [4] (Fig. 4B and Supplementary Fig. 4C, D). Downregulation of genes overexpressed in luminal cells such as GATA3 and PRLR in response to OC2 enforced expression was confirmed in luminal B BT-474 cells and in luminal A MCF-7 cells, this time using an inducible system (Fig. 4C and Supplementary Fig. 4E). Of note, the OC2 inducible system was less sensitive to ER inhibition with tamoxifen (Supplementary Fig. 4F).

The basal subtype is well defined by a gene expression signature characterized by strong expression of basal markers such as cytokeratins (KRT4/5/6A/6B/13/14/15/16/17), integrins (ITGA6, ITGB4/6), MET, LYN, etc. [43, 44]. Transcriptional profiling has allowed the identification of a basal B subtype of cells that are less differentiated and have a more mesenchymal-like appearance [45]. This subtype has been designated the mesenchymal cluster or normal-like/claudin-low and overexpresses genes associated with tumor invasive and aggressive features such as up-regulation of VIM, TGFB1 and SERPINE1/2 and cancer stemness such as CD44 expression [43]. Our results show that enforced OC2 expression significantly alters discriminator expression signatures of the basal/luminal and mesenchymal/luminal phenotype (Fig. 4D, E and Supplementary Fig. 4G, H). Up-regulation of basal and mesenchymal markers such as MET, TGFB1 and CD44 in response to OC2 enforced expression was confirmed in both BT-474 and in MCF-7 cell lines (Fig. 4F, G). To further investigate the relationship between OC2 expression and these linages, we next analyzed scRNA-Seq data from a cohort of 26 primary pre-treatment tumors that include 11 ER positive, 5 HER2 + and 10 basal-like/TNBC [31]. OC2 overlapped with the basal-like/TNBC and HER2 + phenotypes (Fig. 4H) and its expression is significantly higher in ER negative cells compared with ER positive cells (Fig. 4I). Consistent with a role of OC2 in the luminal-to-basal reprogramming and in tumor progression, OC2 mRNA is significantly higher in basal tumors compared to luminal A and B tumors (Fig. 4J and Supplementary Fig. 4I). Of note, OC2 mRNA expression is highest in the HER2 + subtype (Fig. 4J and Supplementary Fig. 4J) suggesting that OC2 might be active in a broader group of hormone receptor-negative aggressive BC tumors with poor prognosis. The analysis of OC2 RNAi Screening using DepMap [32] shows that basal and HER2 + cells exhibit a higher dependency in OC2 than luminal BC cell lines (Supplementary Fig. 4K).

### OC2 acts as a survival factor in BC and it can be targeted with a drug-like molecule

**Fig. 5:**
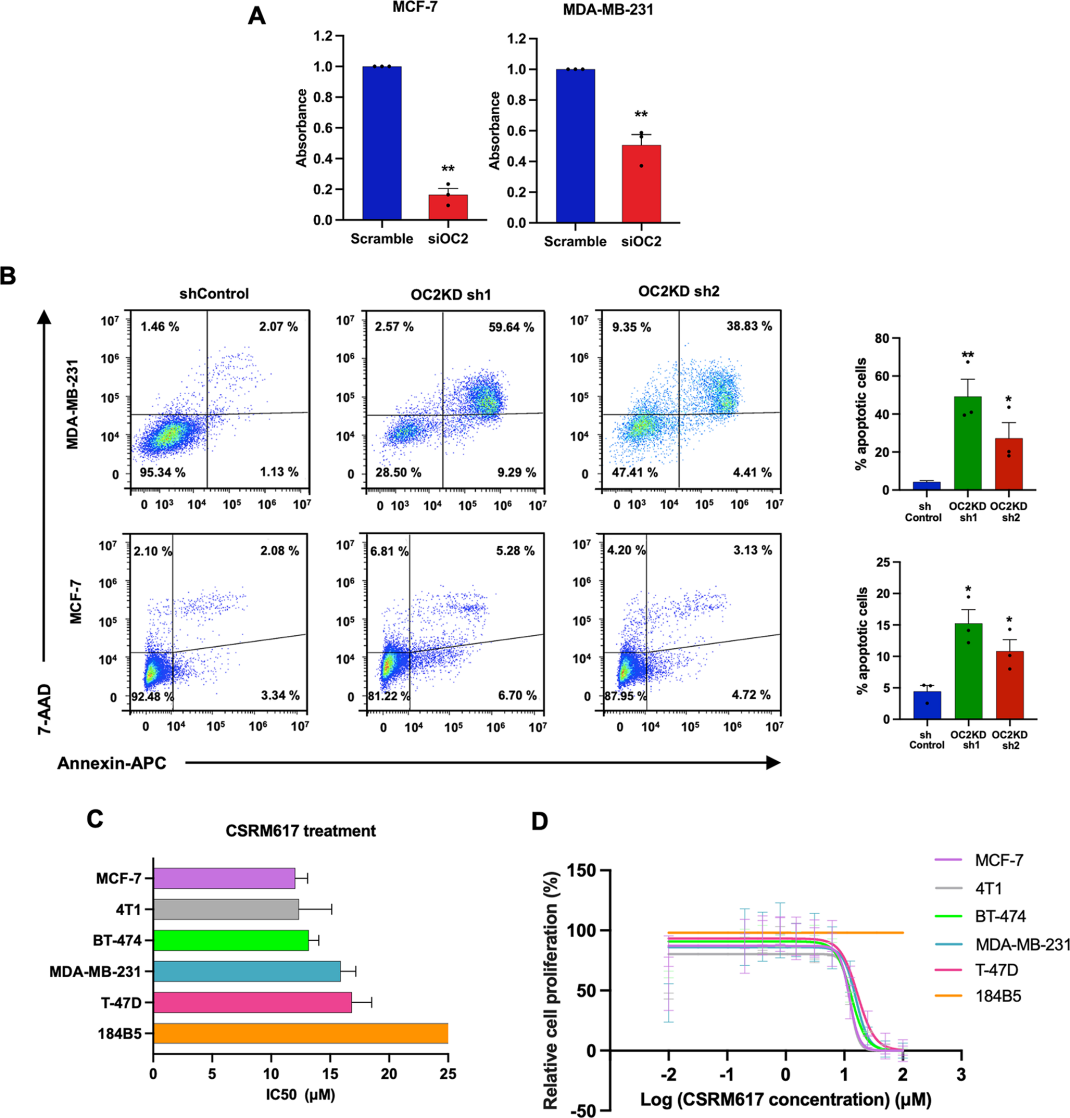
OC2 is required for BC proliferation and survival. **A** Cell viability 2 days after start of OC2 depletion assessed by crystal violet staining. Absorbance levels of MCF-7 (left) and MDA-MB-231 (right) cell lines in control cells (cells transfected with a mixture of non-specific oligonucleotides) and siOC2 (cells transfected with a mixture of oligonucleotides targeting OC2) were measured at 595 nm. The mean + S.E.M from three independent experiments is shown. Unpaired two-tailed Student’s t-test, **P < 0.01. **B** OC2 depletion with shRNAs results in cell death measured by flow cytometry and double staining with Annexin V-APC and 7AAD. The graph shows the mean + S.E.M from three independent experiments. Unpaired two-tailed Student’s t-test, *P < 0.05, **P < 0.01. **C** IC50 values for compound CSRM617 in mouse and human breast cell lines after 48 h treatment. The values shown are the mean + S.E.M from three independent experiments. **D** Dose-response curves of the six breast cell lines to CSRM617. Growth was measured in triplicates and viability was assayed 48 h after treatment. The values shown are the mean ± S.E.M from three independent experiments

To evaluate the function of OC2 in BC we depleted OC2 expression in different BC cell lines. OC2 silencing with siRNA oligos caused loss of viability in luminal MCF-7 and in triple-negative MDA-MB-321 cells (Fig. 5A). Furthermore, OC2 depletion by lentiviral infection with two independent shRNA caused extensive apoptosis in both cell lines (Fig. 5B and Supplementary Fig. 5A). These results suggest that OC2 might be a clinically relevant therapeutic target in the context of BC. Treatment with a small molecule inhibitor of OC2, compound CSRM617 [15], inhibited cell growth in several BC cell lines, including the highly aggressive mouse 4T1 triple-negative tumor model that expresses relatively high OC2 levels (Fig. 5C, D and Supplementary Fig. 5B), phenocopying the effects observed when OC2 was depleted with shRNA or siRNA (Fig. 5A, B). Importantly, nontumorigenic 184B5 cells were resistant to treatment with CSRM617 (Fig. 5C, D).

**Fig. 6:**
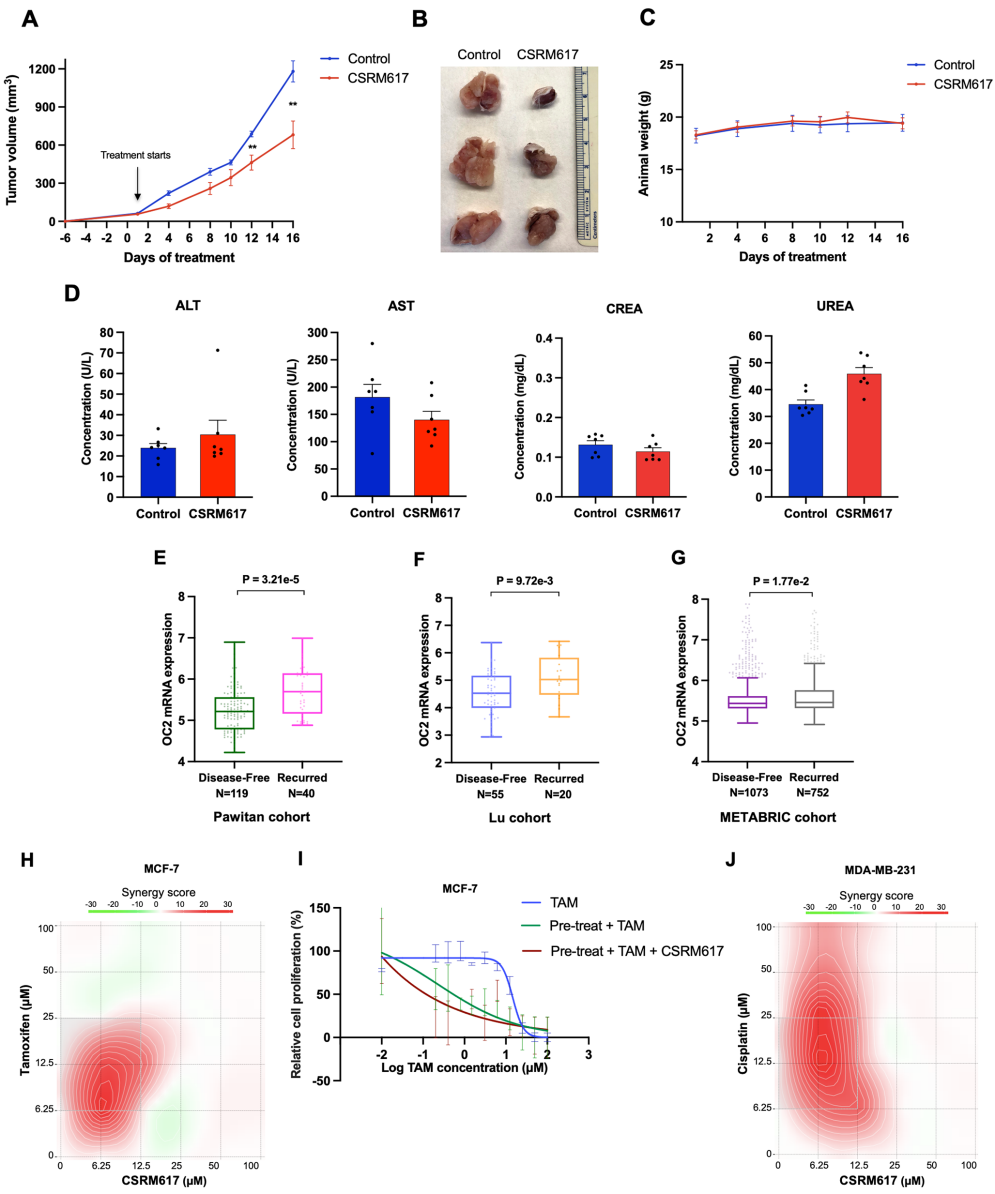
Targeting OC2 inhibits BC growth in mice. **A** 4T1 cells were implanted subcutaneously in the flank of BALBc mice. When tumors reached 50 mm^3^, mice were randomized and received 50 mg kg^− 1^ CSRM617 or vehicle (Control *n* = 7, CSRM617 *n* = 7). The mean ± S.E.M is shown. Unpaired two-tailed Student’s t-test, **P < 0.01. **B** Appearance of tumors after 16 days treatment. **C** CSRM617 treatment did not affect animal weight (mice from A). **D** Liver (ALT and AST) and renal (CREA and UREA) toxicity assessments in serum from mice after CSRM617 treatment. The mean + S.E.M is shown. ALT = alanine aminotransferase; AST = aspartate aminotransferase; CREA = creatinine. **E**–**G** Boxplot of OC2 mRNA expression in three BC cohorts [38, 46, 47] showing significant differences in tumors from disease-free patients compared to patients with recurrent disease. For (**E**, **F**) the boxes show the 25–75th percentile range and the center line is the median. Whiskers extend from the minimum and maximum values. For (**G**) the boxes show the 25–75th percentile range and the center line is the median. Whiskers show 1.5 times the IQR from the 25th or 75th percentile values. Wilcoxon two-tailed rank-sum test *P*-values are shown. For every comparison, *P* < 0.05 was considered significant. **H** Heatmap of combination responses for compound CSRM617 and tamoxifen in MCF-7 cells according to ZIP model. **I** Tamoxifen dose-response curves in the presence or absence of 10 µM CSRM617 in MCF-7 pre-treated cells with 10 µM CSRM617 for 48 h. The values shown are the mean ± S.E.M from at least two independent experiments. **J** Heatmap of combination responses for compound CSRM617 and cisplatin in MDA-MB-231 cells according to ZIP model. For (**H**, **J**) the synergy score was calculated using SynergyFinder software [33]. Positive (red) or negative (green) synergy scores indicate synergistic and antagonistic effects, respectively. The values shown are the mean from two independent experiments

To test the effect of CSRM617 *in vivo*, 4T1 cells were implanted subcutaneously in female BALB/c mice. When tumors reached a size of approximately 50 mm^3^, mice were randomized and treated daily with CSRM617 or vehicle control (2.5% DMSO in PBS) intraperitoneally at a dose of 50 mg kg^− 1^ d^− 1^. The treatment group showed significant reduction of tumor volume (Fig. 6A, B). CSRM617 did not affect body weight and did not alter markers of kidney and liver damage (Fig. 6C, D), suggesting that the compound is overall well tolerated and non-toxic.

Supporting the potential of OC2 as a therapeutic target in aggressive BC disease, in three separate cohorts [38, 46, 47] OC2 mRNA expression is significantly higher in BC specimens from patients that exhibited recurrence compared to patients that remained disease-free (Fig. 6E–G). OC2 mRNA expression is also significantly increased in tamoxifen-resistant cells compared to tamoxifen-sensitive cells, both in 2D monolayers and 3D spheroids cultures [48, 49]. To further investigate whether compound CSRM617 can enhance the tamoxifen sensitivity of luminal BC cells, we exposed MCF-7 cells to CSRM617 and tamoxifen in a serial concentration and examined the effects on cell viability. Figure 6H shows that the combination of compound CSRM617 and tamoxifen exerts synergistic cytotoxic effects in MCF-7 cells. This cell line also shows attenuated cell viability when cells are pre-treated with CSRM617 for 48 h followed by treatment with tamoxifen alone (Fig. 6I). Of note, the IC50 shift is highest when concomitant exposure of both agents follows CSRM617 pre-treatment (Fig. 6I). Finally, we analyzed the combined effect of chemotherapy and OC2 inhibition. The synergistic effect of cisplatin and CSRM617 in MDA-MB-231 cells is shown in Fig. 6J.

## Discussion

Phenotypic plasticity has emerged as a major contributor to BC heterogeneity and treatment resistance in cancer. Our study provides the first evidence that the transcription factor OC2 is a key regulator of tumor cell plasticity and phenotypic heterogeneity allowing adaptation of cells during tumor initiation and progression and driving transitions towards treatment-resistant states. We also show the therapeutic potential of targeting OC2 in BC with a drug-like small molecule.

OC2 has been shown to be a lineage plasticity driver that suppresses the AR transcriptional program by direct regulation of the AR, its target genes and the pioneer factor FOXA1, and activates genes associated with neural/neuroendocrine differentiation and progression to lethal disease [15]. Although prostate and breast cancers arise in organs that are distinct in kind, both organs require gonadal steroids for their development and both tumors are typically hormone-dependent and exhibit remarkable biological similarities [50]. Androgen and estrogen action is mediated by steroid/nuclear receptor-transcription factors (AR and ERα/β) that are therapeutic targets for prostate and breast cancer. Our previous [15] and current results show that OC2 is a suppressor of both oncogenic drivers. This study also shows that PR expression, an important prognosis biomarker in BC, is significantly repressed by OC2 [51] and points to the possibility that OC2 may be a master regulator of steroid receptors in hormone-dependent cancers.

Acquired independence to initial oncogenic drivers is part of the process of lineage plasticity in which tumor cells experience shifts in identity adopting a therapy resistant phenotype and becoming more aggressive [52]. Our results in this study show that luminal BC cell lines that ectopically express OC2 not only see their ER transcriptional program appeased but acquire a basal-like state at the gene expression level. Collective migration of BC has been shown to involve leader cells with a reactivated basal program [53] and to be critical for a successful establishment of metastasis. Interestingly, when analyzing the OC2 cistrome published in Rotinen et al. [15], we find that OC2 binds to promoters in 9 out of 15 genes in a gene expression signature predictive of BC metastasis to bone (Supplementary Fig. 6A) [54]. Evaluation of other BC metastasis signatures to brain and bone [4] demonstrates high enrichment of genes directly regulated by OC2 (28 out of 90 genes downregulated in brain-relapse; 24 out of 98 genes upregulated in bone-relapse). These data suggest that OC2 may be an important driver of BC metastasis and may play a role in determining cell behavior with regard to the sites of distant metastasis. This hypothesis should be tested in future studies.

In BC our study demonstrates that OC2 acts as a survival factor. OC2 gene silencing *in vitro* effectively suppressed proliferation and induced apoptosis of a series of human and mouse BC cell lines representative of the luminal and triple-negative subtypes. Consistent with our findings, in MCF-7 xenograft mice, OC2 silencing with short hairpin RNAs has been shown to exert antitumor effects reducing tumor burden and promoting apoptosis [55]. Importantly, in this study we also show that blocking OC2 activity with a drug-like small molecule reduces tumor growth of extremely aggressive and metastatic 4T1 cells. Because OC2 can be inhibited pharmacologically our results suggest that OC2 may constitute a novel druggable target to TNBC, the molecular subtype with the worst prognosis and limited therapeutic options [56]. OC2 suppression may serve as well to inhibit the emergence of basal-like features arising from phenotypic plasticity and loss of lineage confinement in BC tumors, where it could improve treatment outcomes solo or in combination with other known cancer drugs.

In summary, we have demonstrated that OC2 plays a significant role in the progression of BC from luminal to aggressive hormone-receptor negative forms, making it a potentially attractive and novel therapeutic target. We have also demonstrated that OC2 can be inhibited with a small molecule that reduces tumor growth in a highly aggressive TNBC xenograft model and can sensitize luminal BC cells to tamoxifen. Our study supports the conclusion that many BC tumors, marked by high levels of heterogeneity, consist of cells with loss of luminal differentiation and activation of a basal/mesenchymal program in which OC2 plays a major role. Because OC2 is expressed at all BC subtypes, OC2-targeted therapies hold promise for effectively treating early as well as advanced/drug-resistant forms of BC.

## Electronic supplementary material

Below is the link to the electronic supplementary material.

Supplementary Material 1

## Data Availability

Data generated or analyzed during this study are included in this published article (and its supplementary information files). RNA-seq data generated in this study were deposited in GEO (GSE242541).
