# Supplementary material for: ONECUT2 is a druggable driver of luminal to basal breast cancer plasticity
Source: Cell Oncol (Dordr). 2024 May 31;48(1):83–99. doi: 10.1007/s13402-024-00957-3 (PMC11850477; doi:10.1007/s13402-024-00957-3)
Supplement: Supplementary file 1 — Supplementary Material 1 [file 13402_2024_957_MOESM1_ESM.docx]

**Supplementary Figure 1 – Related to Figure 1.**

**A-D)** Association of relapse-free survival **(A-C)** and overall survival **(D)** with OC2 expression (probes: 239911_at, 233446_at and 230271_at). Patients were divided according to OC2 mRNA expression levels using the median as cut-off. **E-H)** Association of overall survival **(E)** and relapse-free survival **(F-H)** with OC2 expression (probes: 239911_at, 233446_at and 230271_at) in luminal A tumors according to St. Gallen classification. For **(E)** patients were divided according to OC2 mRNA expression levels using Q1 vs Q4 quartiles as cut-off. For **(F-H)** patients were divided according to OC2 mRNA expression levels using the median as cut-off. For **(A-H)** Log-rank Test. For every comparison, P < 0.05 was considered significant.

**Supplementary Figure 2 – Related to Figure 2.**

**A)** OC2 mRNA expression in invasive ductal carcinoma (IDC), invasive lobular carcinoma (ILC), breast mixed ductal and lobular carcinoma (Mixed) and breast invasive mucinous carcinoma (Mucinous) from the METABRIC cohort. The boxes show the 25-75^th^ percentile range and the center line is the median. Whiskers show 1.5 times the interquartile range (IQR) from the 25th or 75th percentile values. Kruskal-Wallis test P-value is shown. P < 0.05 was considered significant. **B)** OC2 mRNA expression and its association with grade from the TCGA BC cohort. **C)** The promoter methylation of OC2 and its association with grade from the TCGA BC cohort. For **(B, C)** the boxes show the 25-75^th^ percentile range and the center line is the median. Whiskers show 1.5 times the interquartile range (IQR) from the 25th or 75th percentile values. Kruskal-Wallis and Dunn’s tests P-values are shown. For every comparison, P < 0.05 was considered significant.

**Supplementary Figure 3 – Related to Figure 3.**

**A)** Immunoblot showing endogenous OC2 levels in human BC cell lines. Representative blots from three independent experiments. **B)** OC2 mRNA expression levels in human BC cell lines. qRT-PCR results were normalized using β-actin. The mean + S.E.M. from three independent experiments is shown. **C)** GSEA plots of three estrogen activity metasignatures [40] showing enrichment of ER target genes in MCF-7 (left) and BT-474 (right) cells that overexpress OC2. Three independent RNA-Seq experiments were performed. **D)** MYC, GREB1, PDZK1 and TFF1 mRNA levels in T-47D cells with enforced OC2 expression, treated for 24 h with 10 nM E2 or vehicle. The mean + S.E.M. from three independent experiments is shown. Unpaired two-tailed Student’s t-test was used for statistical analysis, **=P<0.01. **E, F)** The promoter methylation of OC2 and its association with the ER status from the **(E)** TCGA and **(F)** METABRIC cohorts. For **(E, F)** the boxes show the 25-75^th^ percentile range and the center line is the median. Whiskers show 1.5 times the interquartile range (IQR) from the 25th or 75th percentile values. Wilcoxon two-tailed rank-sum test P-values are shown. For every comparison, P < 0.05 was considered significant.

**Supplementary Figure 4 – Related to Figure 4.**

**A)** Inverse correlation between OC2 expression and ESR1 (left), PGR (middle) and GATA3 (right) expression in the METABRIC cohort. **B)** Inverse correlation between OC2 expression and ESR1 (left), PGR (middle) and GATA3 (right) expression in the TCGA cohort. **C)** GSEA plot showing negative enrichment of a luminal B signature [4] in MCF-7 and BT-474 cells with enforced OC2 expression. Three RNA-Seq experiments were performed per condition. **D)** GSEA plot showing de-repression of down-regulated genes in a luminal B signature [4] in MCF-7 cells with enforced OC2 expression. Three RNA-Seq experiments were performed per condition. **E)** Immunoblots showing the results of enforced expression (OE) of OC2 in BT-474 and MCF-7 cells. Representative blots from three independent experiments. Dox = doxycycline. **F)** IC50 values obtained from dose-response curves to tamoxifen in MCF-7 with enforced OC2 expression using the doxycycline-regulated system. The mean + S.E.M. from six independent experiments is shown. Unpaired one-tailed Student’s t-test was used for statistical analysis, *=P<0.05. **G)** GSEA plot showing negative enrichment of genes up-regulated in luminal-like breast cancer cell lines compared to the mesenchymal-like ones [44] in MCF-7 and BT-474 cells with enforced OC2 expression. **H)** GSEA plot showing positive enrichment of genes down-regulated in luminal-like breast cancer cell lines compared to the mesenchymal-like ones [44] in MCF-7 cells with enforced OC2 expression. For **(G, H)** three RNA-Seq experiments were performed per condition. **I)** The boxplot shows OC2 mRNA expression in different BC subtypes. Data obtained from the Lu cohort [46]. The boxes show the 25-75^th^ percentile range and the center line is the median. Whiskers extend from the minimum and maximum values. ANOVA and Tukey tests P-values are shown. **J)** Expression of OC2 mRNA in HER2-negative vs. HER2-positive patients in the TCGA cohort. The boxes show the 25-75^th^ percentile range and the center line is the median. Whiskers show 1.5 times the IQR from the 25^th^ or 75^th^ percentile values. Wilcoxon two-tailed rank-sum test P-value is shown. For every comparison, P < 0.05 was considered significant. **K)** OC2 dependency score of BC cell lines using DepMap [32]. RNAi screening data grouped by legacy molecular subtype.

**Supplementary Figure 5 – Related to Figure 5.**

**A)** Immunoblot showing the results of OC2 knockdown (KD) in MCF-7 and MDA-MB-231 cells. Representative blots from three independent experiments. **B)** Immunoblot showing endogenous OC2 levels in the mouse BC cell line 4T1 and in a series of human BC cell lines. Representative blots from three independent experiments.

**Supplementary Figure 6 – Related to Discussion**

OC2 binding to BC metastasis associated genes. Chip-Seq OC2 data obtained from Rotinen *et al.* [15].

**Supplementary Figure 7 – Related to Material and Methods**

Representative images of low **(A)**, intermediate **(B)**, and high **(C)** OC2 expression in breast carcinomas contained on the TMA. Scale bar 75 µm.

**Supplementary Table 1. List of the top 100 repressed genes (|log FC| ≥ 1.5 and adjusted P-value < 1e-10) ranked by the adjusted P-value in the MCF-7 RNA-Seq dataset (OC2-OE vs Control)**

**Supplementary Table 2. List of the top 100 repressed genes (|log FC| ≥ 1.5 and adjusted P-value < 1e-10) ranked by the adjusted P-value in the BT-474 RNA-Seq dataset (OC2-OE vs Control)**

**Supplementary Table 3. Top-5 negatively correlated proteins with OC2 (TCGA cohort)**
